# Mental health and resilience among Eritrean refugees at arrival and one-year post-registration in Switzerland: a cohort study

**DOI:** 10.1186/s13104-021-05695-5

**Published:** 2021-07-22

**Authors:** Afona Chernet, Nicole Probst-Hensch, Véronique Sydow, Daniel H. Paris, Niklaus D. Labhardt

**Affiliations:** 1 Swiss Tropical and Public Health Institute, P.O. Box CH-4002, Basel, Switzerland; 2 University of Basel, Basel, Switzerland; 3 Division of Infectious Diseases and Hospital Epidemiology, University Hospital Basel, Basel, Switzerland

**Keywords:** Asylum-seekers, Eritrea, Migration, Refugees, Resilience, PTSD, Mental Health, Switzerland

## Abstract

**Objective:**

Eritrea is the most frequent country of origin among asylum seekers in Switzerland. On their journey through the desert and across the Mediterranean Sea, Eritrea refugees are often exposed to traumatizing experiences. The aim of this study is to assess the mental health status and resilience of Eritrean migrants in Switzerland upon arrival and one-year post-arrival, using standardized mental health screening and resilience assessment tools.

**Results:**

At baseline, 107 refugees (11.2% female, median age 25) were interviewed: 52 (48.6%) screened positive for Post-Traumatic Stress Disorder (score ≥ 30), 10.3% for anxiety (≥ 10) and 15.0% for depression (≥ 10); 17.8% scored as risk/hazardous drinkers (≥ 8). The majority (94.4%) had a high resilience score (≥ 65). For one-year follow-up, 48 asylum seekers could be reached. In interviews 18 (38%) of these reported imprisonment in a transit country and 28 (58%) that they had witnessed the death of a close person along the migration route. At the one year assessment, rates of risky/hazardous alcohol use remained unchanged, rates of positive PTSD screening tended to be lower (50.0% (24/48) at baseline vs 25.0% (12/48) at follow-up), as were rates of positive screening for anxiety (8.3% vs 4.2%) and depression (14.6 vs 6.3%).

**Supplementary Information:**

The online version contains supplementary material available at 10.1186/s13104-021-05695-5.

## Introduction

In Switzerland, Eritreans have formed the largest group of asylum seekers for the last decade. In 2018 alone, out of the 15,255 refugees who sought asylum in the country, 18.5% were Eritreans, followed by refugees from Syria (9.1%) [1].

Many refugees from Eritrea are at risk of being exposed to mentally traumatizing events, be it in their country of origin, or during the migration journey to Europe [2, 3]. Such mental trauma could potentially predispose migrants to develop mental health problems during migration, at or after arrival in their host country. Further, several of the macro and micro root causes of migration also contribute to the wellbeing and mental health status of migrants [4]. Moreover, migration by itself is a frequent cause of impaired mental health [5].

Psychiatric problems constitute a substantial burden to migrants, both during the migration route and post-migration [6–8]. The prevalence of post-traumatic stress disorder (PTSD) among migrants has been reported to be 47% [9]. *Médecins Sans Frontières* reports that 31% and 20% of newly arrived African refugees coming from Libya and landing in Italy had PTSD and/or depression, respectively [10]. A qualitative study conducting in-depth interviews with 10 Eritrean asylum seekers and refugees in Switzerland revealed that somatic and mental health problems are often overlapping and perceived as an entity. Thus, mental health conditions may be underdiagnosed in host countries [11].

In this manuscript, we provide data from mental health screening and interviews among Eritrean asylum seekers and refugees shortly after arrival in Switzerland and at the one-year follow-up.

## Main text

### Methods and materials

#### Participants

The study was conducted in refugees’ centers of two cantons (canton Basel-Land and Basel-Stadt) in the northwestern part of Switzerland. Recently arrived (arrivals in 2014 and 2015) refugees and migrants from Eritrea, who were at least 16 years old and who had no major medical complaints, were invited to take part in the study. The recruitment period was from February 2016 to November 2016. Study participants were approached through the registers provided by the cantonal social assistant offices. Invitation letters written in Tigrigna (an Eritrean local language) and English were sent by mail to potential participants. Refugees and asylum seekers, who were interested in taking part in the study, were briefed on the phone, and invited to the outpatient department of the Swiss Tropical and Public Health Institute (Swiss TPH).

This study is part of a larger study that involved an extensive health check-up, including screening for infectious diseases, cardio-vascular risk factors, and mental health-related problems on arrival and at one-year follow-up. The detailed recruitment procedure and results for infectious diseases and cardio-vascular risk-factor screening have previously been described elsewhere [12]. In this manuscript, we present the data on mental health and resilience screening at arrival and one year follow-up. All participants provided written informed consent for enrollment.

#### Data collection

Interviews took place in a closed room to ensure confidentiality and were conducted in Tigrigna by the first author. Four standardized questionnaires were applied: For alcohol use, the *Alcohol Use Disorders Identification Test* (AUDIT) [13], with a scale from 0–40 was used. To screen for depression, anxiety and somatic problems, the *Patient Health Questionnaire Somatic Anxiety and Depression Syndrome* (PHQ-SADS) package was used [14]. The package is composed of *Patient Health Questionnaire-15* (PHQ-15), *Generalized Anxiety Disorder-7* (GAD-7), and *Patient Health Questionnaire-9* (PHQ-9). The *Post-Traumatic Stress Disorder Check List-Civilian Version* (PTSD-CL-S) was used to screen for PTSD [15], and the *14-Item Resilience Scale* (RS-14) [16, 17] to measure coping and resilience ability.

One year after baseline screening, participants were contacted and invited to a follow-up assessment using the same questionnaires and interview procedures plus an additional questionnaire on experiences during migration and current status of integration in Switzerland.

#### Analysis

All data were collected on paper-based forms and subsequently entered into spreadsheets and EpiInfo version 7 (Centers for Disease Control and Prevention, CDC, 1600 Clifton Road Atlanta, USA) using double data entry. Continuous variables are reported as median with inter-quartile range (IQR); prevalence is reported as percentage with 95% confidence intervals (95%CI). Due to small numbers at follow up, analysis is descriptive. Reporting in this manuscript follows the STROBE guidelines for cross-sectional studies (https://www.strobe-statement.org).

#### Ethics statement

The study protocol was reviewed and approved by the institutional research commission and ethical review board of the Swiss Tropical and Public Health Institute (Swiss TPH, Basel, Switzerland; reference no. FK 120; approval date: June 24, 2015), and the national ethics committee of Northwest and Central Switzerland (reference no. EKNZ 2015-353; approval date: November 20, 2015). Participation was voluntary, and hence, people could withdraw from the study at any time without further obligations. Data were processed anonymously.

### Results

In total, 107 participants were recruited. Table 1 summarizes participants’ characteristics. The majority were males (89%), and the median age was 26 and 23 years in men and women respectively (Table 1).

**Table 1:** Demographic information of Eritrean refugees recruited for the study, at baseline

	Female (N = 12)	Male (N = 95)	Total (N = 107)
Median age (IQR*)	23 (IQR: 19–28)	26 (IQR: 19–32)	25 (IQR: 21–29)
Marital status			
Single	7 (58.3%)	66 (69.5%)	73 (68.2%)
Married	4 (33.3%)	29 (30.5%)	33 (30.8%)
Divorced	1 (8.3%)	0 (0%)	1 (0.9%)
Educational background			
Primary	4 (33.3%)	40 (42.1%)	44 (41.1%)
Secondary	5 (41.7%)	46 (48.4%)	51 (47.7%)
Tertiary	3 (25.0%)	9 (9.5%)	12 (11.2%)
Smoking history			
Smoker	0 (0%)	20 (21.1%)	20 (18.7%)
Ex-smoker	0 (0%)	4 (4.2%)	4 (3.7%)
Non-smoker	12 (100%)	71 (74.7%)	83 (77.6%)
Employment			
Temporary	1 (8.3%)	1 (1.1%)	2 (1.9%)
Unemployed	11 (91.7%)	94 (98.9%)	105 (98.1%)

IQR* = Interquartile range

Table 2 summarizes results of the mental health screening at enrolment. Nineteen (17.8%) had an AUDIT score ≥ 8, indicating risk/hazardous alcohol consumption according to the thresholds recommended by the World Health Organization (WHO). Applying recommended thresholds, the prevalence of positive screening was 15% (16/107) for depression, 10% (11/107) for anxiety, and 10% (11/107) for somatic disorders. For PTSD, 49% (52/107) screened positive. The majority had moderate to high resilience scores on RS-14 (94%; 101/107).

**Table 2:** Mental health screening results at baseline (N = 107)

Screening for	Tests	Cut-off	Interpretation	Baseline (N = 107)		
				Score (N: %)		Median (IQR*)
		0	No risk	40	37.4	NA
		1 to 7	Low risk	48	44.9	3 (2–5)
Alcohol use	AUDIT^1^	8 to 15	Risk/ hazardous	18	16.8	11 (9–13)
		16 to19	High risk/ harmful	1	0.9	NA
		> 20	Almost certainly alcohol dependent	0	0	NA
Somatic symptoms	PHQ-15^2^	≥ 10		11	10.3	11 (11–12)
Anxiety disorder	GAD-7^3^	≥ 10	Moderate and above ^‡^	11	10.3	10 (10–11)
Depression	PHQ-9^4^	≥ 10		16	15.0	11 (11–13)
Post-trauma stress disorder	PTSD^5^	≥ 30	Symptoms of PTSD	52	48.6	35 (31–39.5)
		< 65	Low resilience	6	5.6	59.5 (56–60)
Resilience scale	RS-14^6^	65 to 80	Moderate resilience	37	34.6	76.0 (73–78)
		≥ 81	High resilience	64	59.8	88.0 (86–92)

AUDIT^1^ = Alcohol use disorders identification test

PHQ-15^2^ = Patient health questionnaire-15

GAD^3^ = Generalized anxiety disorder-7

PHQ-9^4^ = Patient Health quationnaire-9

PTSD^5^ = Post-traumatic stress disorder

RS-14^6^ = Resilience scale-14

IQR^*^ = Interquartile range

^‡^ = This applies for all three screening tools. The cut-offs for mild, **moderate** and severe conditions are 5, **10**, and 15, respectively

Out of the 107 participants enrolled, 48 (45.0%) attended the one-year follow-up screening. The remaining participants had either moved to a different canton or out of Switzerland (45.0%), or declined participation in the follow-up visit (10.0%). Table 3 provides socio-economic characteristics and reported traumatic events during the migration process from these 48 participants. The median duration of the migration journey (from Eritrea to Switzerland) was 8.5 months. Imprisonment during transit to Europe was reported by 38%, and 58% stated having witnessed the death of a close person during migration. One-year post-arrival the majority, 58% still did not have a stable residence status in Switzerland; nearly all lived from social assistance and reported a monthly net income below CHF 1000.

**Table 3:** Potential factors that may influence the mental health condition and resilience of newly arrived refugees and migrants from Eritrea; conditions reported in one year follow-up (N = 48)

Parameters or indicators in follow-up (N = 48)	Responses	N	%
Factors related to self-sustenance			
Current status of residence permit (legal status)^‡^	B	12	25.0
	F	8	16.7
	N	28	58.3
New residence permit within one year	Yes	9	18.8
	No	39	81.3
Working, including unpaid work	Yes	20	41.7
	No	28	58.3
Source of income	Social assistance	46	95.8
	Partial social assistance	2	4.2
Monthly income: in Swiss Francs (CHF)^¥^	< 500.00	26	54.2
	≥ 500 and < 1000	21	43.8
	≥ 1000.00	1	2.1
Factors related to acculturation and settlement in the new host country			
Current language competency level (German)	A1	7	14.6
	A2	7	14.6
	B1	14	29.2
	B2	3	6.3
Access to regular local language classes^§^	Yes	28	58.3
	No	20	41.7
Switzerland as a first choice of destination?	Yes	29	60.4
	No	19	39.6
Other first-choice destination country, if applicable	United Kingdom	12	25.0
	Netherlands	3	6.3
	Norway	2	4.2
	Sweden	1	2.1
	No idea	1	2.1
Relative/s in Switzerland	Yes	27	56.3
	No	21	43.8
Interaction with Eritrean community in Switzerland ^α^	Yes	41	85.4
	No	7	14.6
Community contribution in Switzerland ^β^	Yes	26	54.2
	No	22	45.8
Factors related to pre-arrival in host country			
Migration journey duration (from Eritrea to Switzerland): in months	≥ 3 and < 9	24	50.0
	≥ 9 and < 15	10	20.8
	≥ 15 and < 21	7	14.6
	≥ 21 and < 27	4	8.3
	≥ 27	3	6.3
	*Median (IQR)*^μ^	*8.5*	*7.0–16.0*
Duration of stay in Transit countries: in months *Median and Inter Quartile Range (IQR)*^μ^	Ethiopia	*3*	*1.5–5.0*
	Sudan	*5*	*2.0–9.0*
	Libya	*2*	*1.3–3.0*
	Italy	*0.3*	*0.0*
Detention or prison experience during transit	Yes	18	37.5
	No	30	62.5
Imprisonment in transit countries	Egypt	1	2.1
	Ethiopia	4	8.3
	Libya	11	22.9
	Sudan	2	4.2
Experienced death of someone along the migration route^†^	Yes	28	58.3
	No	20	41.7

^‡^ = Current legal residence status of refugees and migrants; B → Refugee status granted; F → Temporarily admitted; and N → Asylum application in process

^¥^ = Monthly income: in Swiss Francs (CHF), for socially assisted immigrants, solely depends on the asylum status condition

^§^ = Similar to other benefits from the social system, access to regular local language courses, with few exceptional cases, is claimed to be affected by the asylum status of refugees

^α^ = Opportunities to have social interactions with compatriot Eritreans, such as social gatherings and hanging out together

^β^ = Willing to interact with the local population by participating in different activities, such as sports, feasts, and volunteering

^†^ = Could be a family member, relative, friend, or a travel colleague introduced on the journey

^µ^ = Figures in italics donate for Inter Quartile Range (IQR) values

Additional file 1: Table S1 displays the mental health screening results at baseline and at the one-year follow-up in the sub-group with complete one-year follow up data (N = 48). Rates of hazardous alcohol consumption did not change substantially. There was a non-significant trend to improvement in somatic symptoms, anxiety disorder and depression. The prevalence of positive screening for PTSD halved from 50% (24/48) to 25% (12/48). Resilience scores remained high.

### Discussion

This study assessed the mental health status and resilience among Eritrean migrants at arrival in Switzerland and one-year post registration. On arrival, almost half of Eritrean refugees screened positive for PTSD. Positive screenings for other mental health conditions were less frequent. Apart from alcohol use that remained unchanged, the prevalence of positive screening decreased for all other conditions at the one-year follow-up. This change was, however, not statistically significant. In general, Eritrean refugees showed high resilience scores. Interviews with participants revealed that the majority were exposed to psychological harm during their transit to Europe, i.e. imprisonment in one of the transit countries or witnessing the death of a close person. Such events are likely to be the cause of the high prevalence of PTSD at arrival in Europe.

In line with our findings, several studies report close to 50% prevalence of PTSD among migrants and refugees arriving in their host country. However, published studies included no or very few participants from Eritrea or East Africa [9, 13, 18, 19]. As shown in Table 3, a high share of participants in our study has experienced psychological trauma. Similarly, a study investigating traumatic experiences of East African migrants arriving in Israel showed that 56.0 and 34.9% of men and women respectively had been victims of various forms of violence [3].

At baseline as well as one-year post registration, participants had high scores on the resilience scale (Table 2 and Additional file 1: Table S1). The high resilience may be a reason for the reduction of positive PTSD screening at one-year follow-up and the overall rather low prevalence of depression and anxiety disorder in this refugee population [20, 21].

In summary, as shown in other refugee populations, our data demonstrate a high burden of mental health conditions at arrival in the host country, particularly PTSD. Further, even though we observed a tendency to improved mental health status one year post-registration, one out of four participants still screened positive for PTSD. These findings should sensitize health authorities as well as clinicians in host countries to consider screening for mental health conditions among Eritrean refugees.

## Limitations

This study has several limitations, mainly the relatively small sample size of 107 participants, and only 48 participants who could be included in the one-year follow-up analysis. In addition, there is relatively low participation of female refugees. Moreover, while interpreting our findings, one must be aware that they rely on self-reporting and that the thresholds used in the applied questionnaires constitute a positive screening result but not a definite diagnosis of the respective mental health condition.

## Supplementary Information

**Additional file 1: Table S1.** Mental health screening at baseline and one-year follow-up (N = 48).

## Data Availability

The datasets generated and analyzed during the current study are not publicly available due the study is part of a larger cohort study, and the data set is not yet publicly available. But are available from the corresponding author on reasonable request.

## Abbreviations

AUDIT: Alcohol Use Disorders Identification Test
PTSD: Post-Traumatic Stress Disorder
PHQ-SADS: Patient Health Questionnaire Somatic Anxiety and Depression Syndrome
PHQ-15: Patient Heath Questionnaire-15
GAD-7: Generalized Anxiety Disorder-7
PHQ-9: Patient Health Questionnaire-9
