# Supplementary material for: Mental health and resilience among Eritrean refugees at arrival and one-year post-registration in Switzerland: a cohort study
Source: BMC Res Notes. 2021 Jul 22;14:281. doi: 10.1186/s13104-021-05695-5 (PMC8299667; doi:10.1186/s13104-021-05695-5)
Supplement: Supplementary file 1 — Additional file 1: Table S1. Mental health screening at baseline and one-year follow-up (N = 48). [file 13104_2021_5695_MOESM1_ESM.docx]

| **Screening for** | **Tests** | **Cut-off** | **Interpretation** | **Baseline** | | **Follow-up** | |
| --- | --- | --- | --- | --- | --- | --- | --- |
|  |  |  |  | **Scores (N; %)** | | **Scores (N; %)** | |
|  |  | 0 | No risk | 17 | 35.4 | 19 | 39.6 |
|  |  | 1 to 7 | Low risk | 24 | 50.0 | 23 | 47.9 |
| Alcohol use | AUDIT^1^ | 8 to 15 | Risk/ hazardous | 6 | 12.5 | 4 | 8.3 |
|  |  | 16 to 19 | High risk/ harmful | 1 | 2.1 | 1 | 2.1 |
|  |  | ≥20 | Almost certainly alcohol dependent | 0 | 0 | 1 | 2.1 |
| Somatic symptoms | PHQ-15^2^ | ≥10 |  | 5 | 10.4 | 0 | 0 |
| Anxiety disorder | GAD-7^3^ | ≥10 | Moderate and above ^‡^ | 4 | 8.3 | 2 | 4.2 |
| Depression | PHQ-9^4^ | ≥10 |  | 7 | 14.6 | 3 | 6.3 |
| Post-traumatic stress disorder | PTSD^5^ | ≥30 | Symptoms of PTSD | 24 | 50.0 | 12 | 25.0 |
|  |  | <65 | Low resilience | 2 | 4.2 | 3 | 6.3 |
| Resilience scale | RS-14^6^ | 65 to 80 | Moderate resilience | 17 | 35.4 | 17 | 35.4 |
|  |  | ≥81 | High resilience | 29 | 60.4 | 28 | 58.3 |

AUDIT^1^= Alcohol use disorders identification test

PHQ-15^2^= Patient health questionnaire-15

GAD^3^= Generalized anxiety disorder-7

PHQ-9^4^= Patient Health quationnaire-9

PTSD^5^= Post-traumatic stress disorder

RS-14^6^= Resilience scale-14

^‡^ = This applies for all three screening tools. The cut-offs for mild, **moderate** and severe conditions are 5, **10**, and 15, respectively.

**Table S1:** Mental health screening at baseline and one-year follow-up (N=48)
